# In-Plane Mechanical Behavior of a New Star-Re-Entrant Hierarchical Metamaterial

**DOI:** 10.3390/polym11071132

**Published:** 2019-07-03

**Authors:** Wenjiao Zhang, Shuyuan Zhao, Rujie Sun, Fabrizio Scarpa, Jinwu Wang

**Affiliations:** 1 School of Engineering, Northeast Agricultural University, No. 600 Changjiang Road, Harbin 150030, China; 2 Center for Composite Materials and Structures, Harbin Institute of Technology, Harbin 150080, China; 3 Bristol Composites Institute (ACCIS), University of Bristol, Bristol BS8 1TR, UK

**Keywords:** hierarchical, metamaterial, re-entrant structure, auxetic, mechanical behavior

## Abstract

A novel hierarchical metamaterial with tunable negative Poisson’s ratio is designed by a re-entrant representative unit cell (RUC), which consists of star-shaped subordinate cells. The in-plane mechanical behaviors of star-re-entrant hierarchical metamaterial are studied thoroughly by finite element method, non-dimensional effective moduli and effective Poisson’s ratios (PR) are obtained, then parameters of cell length, inclined angle, thickness for star subordinate cell as well as the amount of subordinate cell along *x*, *y* directions for re-entrant RUC are applied as adjustable design variables to explore structure-property relations. Finally, the effects of the design parameters on mechanical behavior and relative density are systematically investigated, which indicate that high specific stiffness and large auxetic deformation can be remarkably enhanced and manipulated through combining parameters of both subordinate cell and parent RUC. It is believed that the new hierarchical metamaterial reported here will provide more opportunities to design multifunctional lightweight materials that are promising for various engineering applications.

## 1. Introduction

Re-entrant honeycomb structures that display negative Poisson’s ratios (NPR) are known to be one class of auxetic structures and have been used in many fields, such as aerospace and automotive industries. The multifunctionality of anisotropic re-entrant honeycomb has been widely studied for its static mechanical behavior [1,2,3,4], dynamic performance [5,6], thermal conductivity and heat transfer properties [7].

Hierarchy [8] is one of the most readily observed topological features in natural structures and now has been introduced to honeycomb and chiral lattice structures in pursuing ultralight materials with improving elastic properties and damage tolerance. Specifically, considering hierarchical sub-structures to honeycombs and designing novel metamaterials with tailorable multi-functional properties, have attracted increasing attention in recent years.

Extreme values of hierarchical metamaterial properties such as specific stiffness, toughness, strength, negative or complex Poisson’s ratio, zero or negative thermal expansion, phononic band gaps as well as impact energy absorption have been reported in hierarchical architectures across multiple length scales [9,10,11,12,13,14,15]. Sun et al. [16] analytically studied the in-plane elastic moduli and thermal conductivity of a multifunctional hierarchical honeycomb (MHH), which is formed by replacing the solid cell walls of an original regular hexagonal honeycomb (ORHH) with three different isotropic honeycomb sub-structures possessing hexagonal, triangular or kagome lattices. Then the anisotropic multifunctional hierarchical honeycomb (AMHH) with triangular or kagome honeycomb substructures (OAHH) was proposed and the in-plane stiffness of these two kinds of AMHH was analytically studied with the help of Euler beam theory [17]. Taylor et al. [18] investigated the in-plane elastic properties and structural hierarchy in honeycombs and explored the effects of adding hierarchy into a range of honeycombs, with hexagonal, triangular or square geometry super and sub-structure cells by using finite element simulation. Key parameters describing these geometries included the relative lengths of the sub- and super-structures, the fraction of mass shared between the sub- and super-structures, the co-ordination number of the honeycomb cells, the form and extent of functional grading, and the Poisson’s ratio of the sub-structure. Mousanezhad et al. [19] studied the effects of chirality and hierarchy on elastic response of honeycombs, derived the closed-form expressions for elastic moduli of several chiral, anti-chiral and hierarchical honeycombs with hexagon and square based networks, and finally validated the analytical estimates of the elastic moduli by using finite element method. Gatt et al. [20] proposed a new class of hierarchical auxetics based on the rotating rigid unit mechanism. These systems retain the enhanced properties from having a negative Poisson’s ratio with the added benefits of being a hierarchical system. Through design, one can control the extent of auxeticity, degree of aperture and size of the different pores in the system, which makes the system more versatile than similar non-hierarchical ones. Chen et al. [21] reported a group of hierarchically architected metamaterials constructed by replacing cell walls of regular honeycombs with hexagonal, kagome, and triangular lattices, respectively. The numerical and analytical studies indicate that the introduction of structural hierarchy in regular honeycombs results in improved heat resistance and thermal anisotropy. Then, Yin et al. [22] studied the in-plane crashworthiness of the hierarchical honeycomb group above, using the nonlinear finite element code LS-DYNA. The numerical simulation results indicate that the triangular hierarchical honeycomb provides the best performance compared to the other two hierarchical honeycombs and features more than twice the energy absorbed by the regular honeycomb under similar loading conditions. More recently, Wu et al. [23,24] proposed an innovative hierarchical anti-tetrachiral structure as well as a hierarchical anti-tetrachiral stent with circular and elliptical nodes, based on the auxetic deformation behaviors of anti-tetrachiral unit cell at different structural hierarchical levels. It was found that the mechanical behaviors of hierarchical anti-tetrachiral structure can be tailored through adjusting the levels of hierarchical structures and unit cell design, and the proposed hierarchical anti-tetrachiral stents exhibit remarkable radial expanding abilities while maintaining axial stability. Besides the above-mentioned hierarchical metamaterials, various types of alternative hierarchical structures have been proposed through the modification of the node or cell wall structural levels for generating enhanced and tunable mechanical properties through structural hierarchy approaches.

In the current work, a novel auxetic hierarchical metamaterial was designed, which consisted of a re-entrant representative unit cell as well as a star subordinate cell with zero Poisson’s ratio; both the main and sub cells were planar symmetric. Full dimensional models of the new hierarchical metamaterial to describe the effective elasticity as well as loading-bearing capability in plane were simulated by finite element method (FEM). Comprehensive parametric studies for both parent RUC and subordinate cell were performed to evaluate in-plane non-dimensional moduli, effective Poisson’s ratio and relative density of the new hierarchical structure, corresponding optimum structure-property relations were explored for the designed metamaterial.

## 2. Materials and Methods

**Figure 1:**
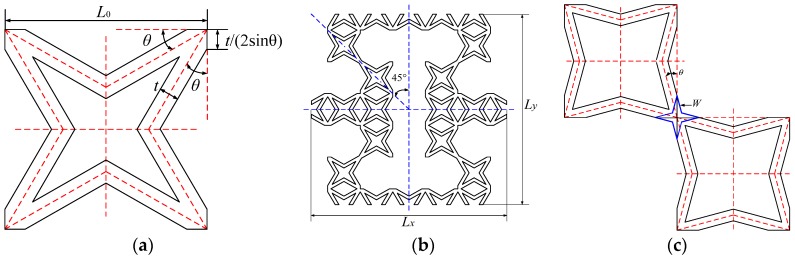
Geometry of the novel star-re-entrant hierarchical metamaterial: (**a**) star subordinate cell, (**b**) re-entrant representative unit cell, (**c**) the jointing between two neighboring stars.

The geometry of the novel hierarchical star-re-entrant metamaterial is presented in Figure 1. The representative unit cell (RUC), composed of star subordinate cell can be described as a re-entrant hexagonal structure, and apparently both the cells are horizontal and vertical symmetry. Analogy to a zero Poisson’s ratio cellular structure [25], the star subordinate cell is represented by length $L_{0}$, inclined angle θ as well as in-plane thickness t, respectively (Figure 1a). Then dimensions of the new hierarchical star-re-entrant RUC is described by length $L_{x}$, $L_{y}$ and symmetric inclined angle $45^{\circ }$ (Figure 1b), where $L_{i}=N_{i}\times L_{0}$ (i=x,y), $N_{i}$ is the number of star subordinate cells along x or y direction. The detail of star joint in re-entrant structure is presented in Figure 1c, the length of jointing star is expressed as $W=\left(\sqrt{2}t\right)/\left(4\sin\theta \cos(45^{\circ }-\theta )\right)$. To avoiding the overlapping and contact of cell walls, geometric constraints $0^{\circ }<\theta <45^{\circ }$ and $0<t<L_{0}\left(\cos\theta -\sin\theta \right)$ need to be satisfied.

The relative density is an important parameter for cellular structures, and it is defined by:

(1) $$\frac{\rho }{\rho_{c}}=\frac{A}{A_{c}}$$

where A and $A_{c}$ are respectively the cross-sections perpendicular to the out-plane thickness direction and the load bearing area. Here, for this new hierarchical RUC, relative density results in:

(2) $$\frac{\rho }{\rho_{c}}=\frac{36L_{0}t\sin3\theta -36L_{0}t\sin\theta -10t^{2}\sin2\theta -8t^{2}\cos2\theta -8t^{2}}{9L_{0}^{2}\sin4\theta }$$

**Figure 2:**
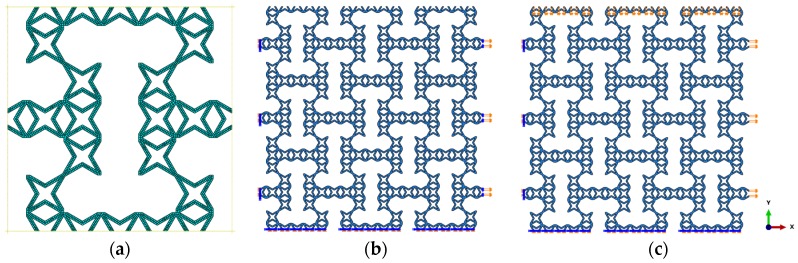
Numerical model description: (**a**) Mesh of one representative unit cell, (**b**) boundary conditions of axial tension along x direction, (**c**) boundary conditions of biaxial shear test.

Finite element analysis was performed with Abaqus/CAE 6.13–4 commercial package standard for one RUC as well as the whole hierarchical metamaterial structure. For all the simulations in this paper, geometric dimensions of the new RUC are defined as $L_{0}=20$, $\theta =30^{\circ }$, t=2 and $L_{x}=L_{y}=6L_{0}$ with symmetric inclined angle $45^{\circ }$, respectively. Acrylonitrile butadiene styrene (ABS) plastic with a rapid prototyping Fusion Deposition Molding (FDM) Stratasys machine was used to manufacture all the experimental samples and the elastic mechanical properties of the core material for finite element simulation were set as $E_{c}=2265\mathrm{MPa}$ and Poisson’s ratio $\nu_{c}=0.25$ [25,26]. An elastic shell element with reduced integration (S4R) and element size of 0.8 for convergence were chosen for the simulation of in-plane effective moduli, shown in Figure 2a. Accounting for the symmetry, the moduli of elasticity and the Poisson’s ratio were determined by one quarter of the metamaterial structure. Taking full-size representative volumes with 6 × 6 cells for example, boundary conditions for in-plane tensile Young’s moduli and Poisson’s ratio were established based on References [23,27], where nodes on the left and bottom edge were constrained from out-plane rotation and translation normal to the edge direction, respectively, and displacements in the x-direction (y-direction) were applied to the ligament nodes on the right (top) edge, which was also constrained from in-plane rotation, shown in Figure 2b. In the case of the in-plane shear simulation, biaxial loading was introduced as close as possible a pure shear deformation field [27,28], corresponding boundary conditions above as well as displacements in both x and y directions were applied and are presented in Figure 2c.

Nominal strain and stress of the hierarchical representative unit cell in i( = *x* or *y*) direction were calculated from:

(3) $$\varepsilon_{i}=\frac{\delta_{i}}{L_{i}},\;\sigma_{i}=\frac{F_{i}}{A_{j}}$$

where $\delta_{i}$ is the applied displacement, $F_{i}$ is the sum of the nodal reaction forces on the edge to which displacement was applied, $L_{i}$ and $A_{j}$ are the initial length and cross-sectional area of the hierarchical structure in the *i* and *j* (= *y* or *x*) directions, respectively. According to Equation (3), effective Young’s modulus as well as Poisson’s ratio are calculated by:

(4) $$E_{i}=\frac{\sigma_{i}}{\varepsilon_{i}},\;\nu_{ij}=-\frac{\delta_{j}L_{i}}{\delta_{i}L_{j}}$$

where, i is the loading direction.

Then, effective shear modulus under biaxial loading in plane was obtained as following [27,28]:

(5) $$G_{xy}=\frac{\tau }{\gamma }=\frac{\sigma_{x}-\sigma_{y}}{2(\varepsilon_{x}-\varepsilon_{y})}=\frac{R_{x}L_{x}-R_{y}L_{y}}{2h(\delta_{x}L_{y}-\delta_{y}L_{x})}$$

In Equation (5), $R_{x}$ and $R_{y}$ are reaction force along x and y direction, h is the out-plane thickness of hierarchical structure.

**Figure 3:**
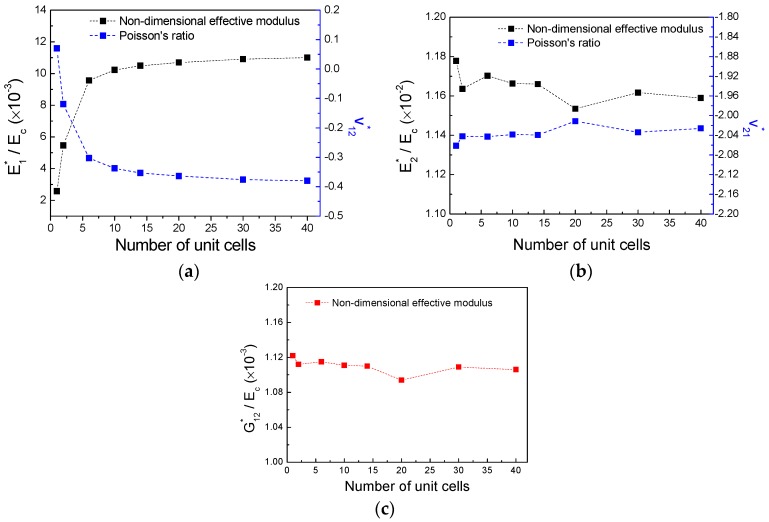
Finite element simulations of effective mechanical property for the new hierarchical metamaterial: (**a**) $E_{1}^{*}/E_{c}$ and $\nu_{12}^{*}$, (**b**) $E_{2}^{*}/E_{c}$ and $\nu_{21}^{*}$, (**c**) $G_{12}^{*}/E_{c}$.

In order to highlight the influence of the cell numbers on the convergence of the results, computations were undertaken starting by a number of 2 × 2 cells to a maximum of 40 × 40 cells. The convergence was found to be achieved at the number of 40 cells; corresponding dependence of mechanical property on the computations number of cells as well as the results of effective non-dimensional moduli and Poisson’s ratio are demonstrated in Figure 3, respectively.

## 3. Results and Discussion

To understand how the geometrical parameters of the star-re-entrant RUC influence the effective mechanical properties of the new hierarchical metamaterial designed, parametric studies were conducted by using finite element models described in Section 3, and the numerical results are presented and discussed as follows.

### 3.1. The Geometry Effects of Star Subordinate Cell

**Figure 4:**
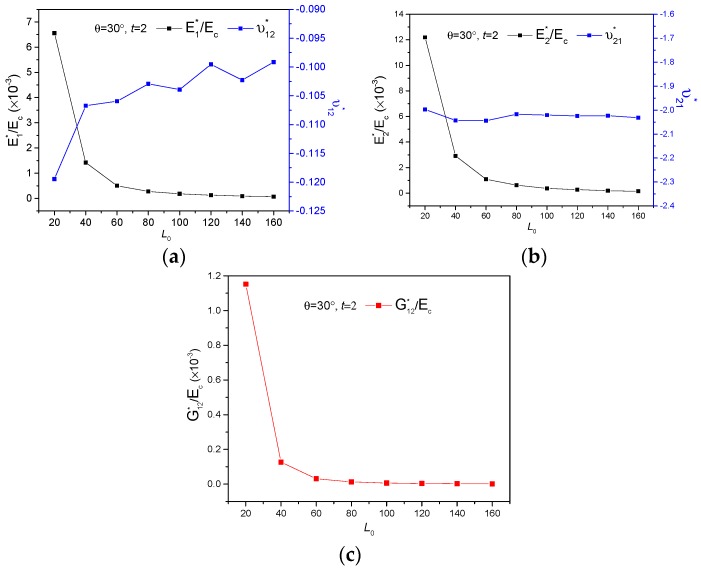
The variations of effective mechanical properties verse length $L_{0}$: (**a**) $E_{1}^{*}/E_{c}$ and $\nu_{12}^{*}$, (**b**) $E_{2}^{*}/E_{c}$ and $\nu_{21}^{*}$, (**c**) $G_{12}^{*}/E_{c}$.

**Figure 5:**
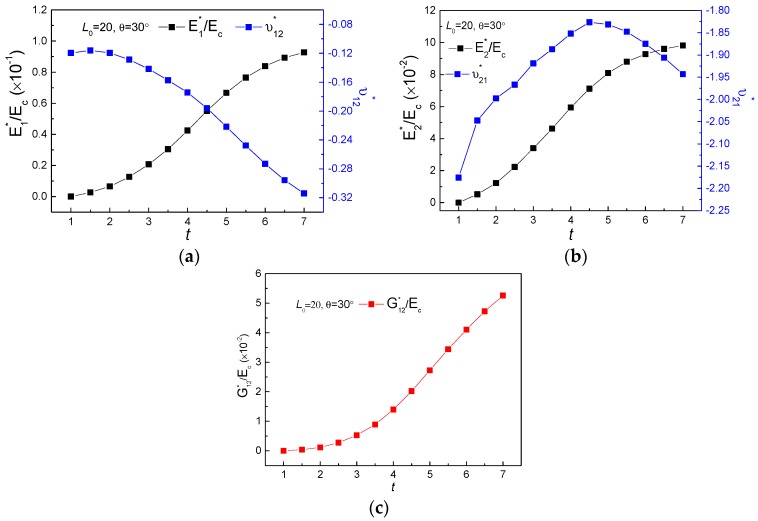
The variations of effective mechanical properties verse thickness t: (**a**) $E_{1}^{*}/E_{c}$ and $\nu_{12}^{*}$, (**b**) $E_{2}^{*}/E_{c}$ and $\nu_{21}^{*}$, (**c**) $G_{12}^{*}/E_{c}$.

**Figure 6:**
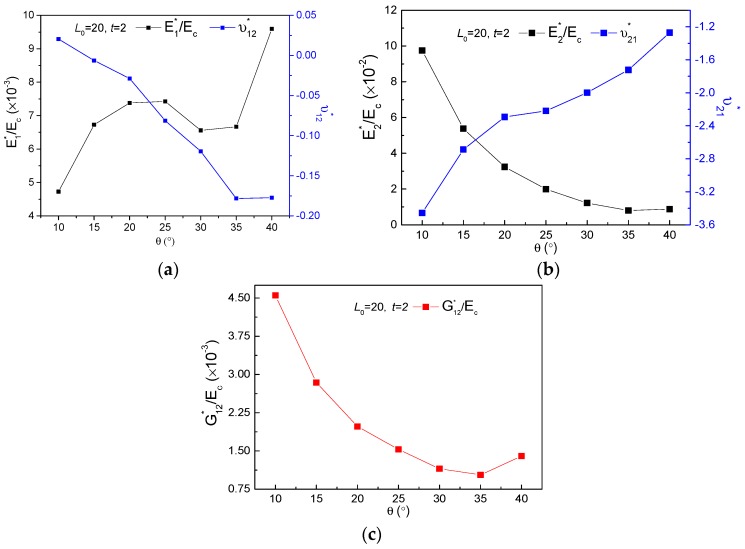
The variations of effective mechanical properties verse inclined angle θ: (**a**) $E_{1}^{*}/E_{c}$ and $\nu_{12}^{*}$, (**b**) $E_{2}^{*}/E_{c}$ and $\nu_{21}^{*}$, (**c**) $G_{12}^{*}/E_{c}$.

Figure 4, Figure 5 and Figure 6 demonstrate the FE homogenization of the non-dimensional in-plane elastic moduli and corresponding Poisson’s ratio versus various parameters of lengths $L_{0}$, thickness t and cell inclined angle θ. In general, all the non-dimensional elastic moduli decrease with increasing $L_{0}$, shown in Figure 4 and increase with increasing thickness t, seen in Figure 5, when the other geometrical parameters keep constant. The variations of non-dimensional effective moduli with cell inclined angle θ are presented in Figure 6. For the increase of θ, $E_{1}^{*}/E_{c}$ exhibits an up-down-up trend, while the other two non-dimensional moduli display a first descent and then ascent with different gradients, for which $E_{2}^{*}/E_{c}$ and $G_{12}^{*}/E_{c}$ increased by 9.89% and 35.9%, respectively. The impact of geometric parameters on effective Poisson’s ratio of the new hierarchical metamaterial are discussed as following. Poisson’s ratio $\nu_{12}^{*}$ only increases with increasing $L_{0}$ (Figure 4a) and declines with thickness t and cell inclined angle θ, presented in Figure 5a and Figure 6a, respectively. While the variations of Poisson’s ratio $\nu_{21}^{*}$ with rising $L_{0}$, θ and t exhibit as constant (Figure 4b), up-down (Figure 5b) as well as ascending (Figure 6b), separately.

**Figure 7:**
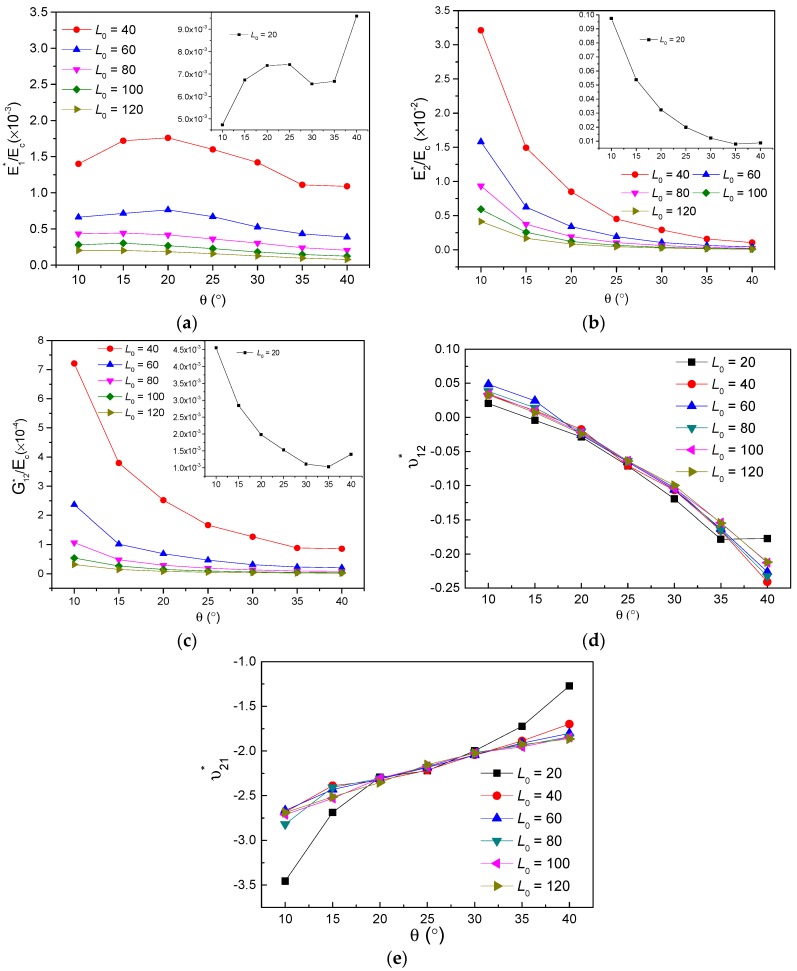
Effects of parameters $L_{0}$ and θ on the effective mechanical property for t=2: (**a**) $E_{1}^{*}/E_{c}$, (**b**) $E_{2}^{*}/E_{c}$, (**c**) $G_{12}^{*}/E_{c}$, (**d**) $\nu_{12}^{*}$, (**e**) $\nu_{21}^{*}$.

In view of increasing cell thickness t mainly enhancing the structural weight and stiffening mechanical behaviors, therefore, the effects of cell inclined angle θ for different $L_{0}$ on in-plane mechanical property were studied in detail and are demonstrated in Figure 7. It may be observed that variation and magnitude of the effective mechanical properties for $L_{0}=20$ with enhancive θ are completely different from those of $L_{0}=40-120$. With the increasing θ, non-dimensional modulus $E_{1}^{*}/E_{c}$ displays an increasing and decreasing trend for $L_{0}=40,60$, and exhibits a gradual decrease for $L_{0}=80-120$, presented in Figure 7a; $E_{2}^{*}/E_{c}$ and $G_{12}^{*}/E_{c}$ are both observed declines with slower slopes, for $L_{0}=40$ to $L_{0}=120$, shown in Figure 7b,c, respectively. For the study of Poisson’s ratio, the increasing cell angle θ makes $\nu_{12}^{*}$ decrease and $\nu_{21}^{*}$ increase inversely, seen in Figure 7d,e. Additionally, parameter $L_{0}$ makes no apparent effect on Poisson’s ratio, except the scenarios of $L_{0}=20$, where $\nu_{12}^{*}$ remains constant in the range of $\theta =35^{\circ }$ to $\theta =40^{\circ }$ as well as $\nu_{21}^{*}$ behaviors a relatively great variation from −3.456 with $\theta =10^{\circ }$ to −1.271 with $\theta =40^{\circ }$.

### 3.2. The Effects of Subordinate Cell Amount

**Figure 8:**
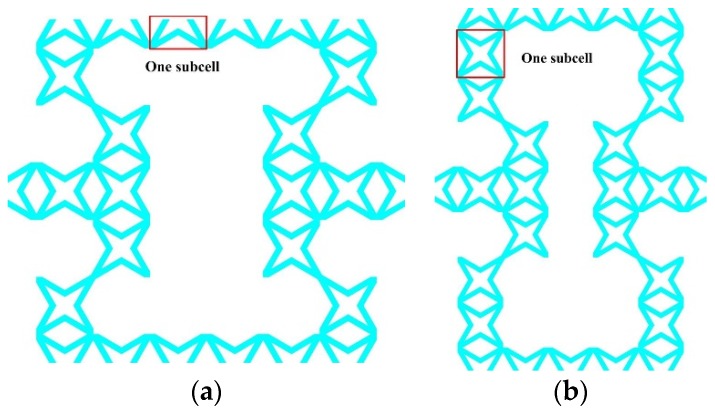
The number variations of star subordinate cell: (**a**) $N_{x}$ along *x* direction, (**b**) $N_{y}$ along *y* direction.

**Figure 9:**
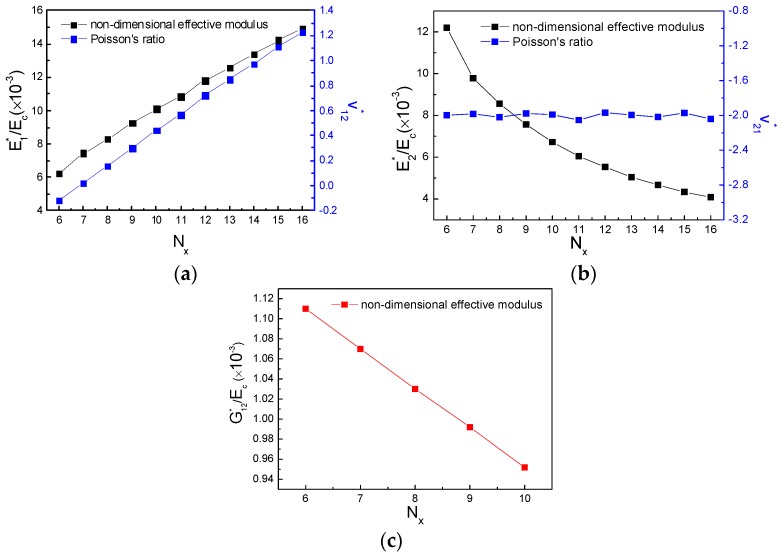
Variation of subordinate cell amount $N_{x}$ verse: (**a**) $E_{1}^{*}/E_{c}$ and Poisson’s ratio $\nu_{12}^{*}$, (**b**) $E_{2}^{*}/E_{c}$ and Poisson’s ratio $\nu_{21}^{*}$, (**c**) $G_{12}^{*}/E_{c}$.

**Figure 10:**
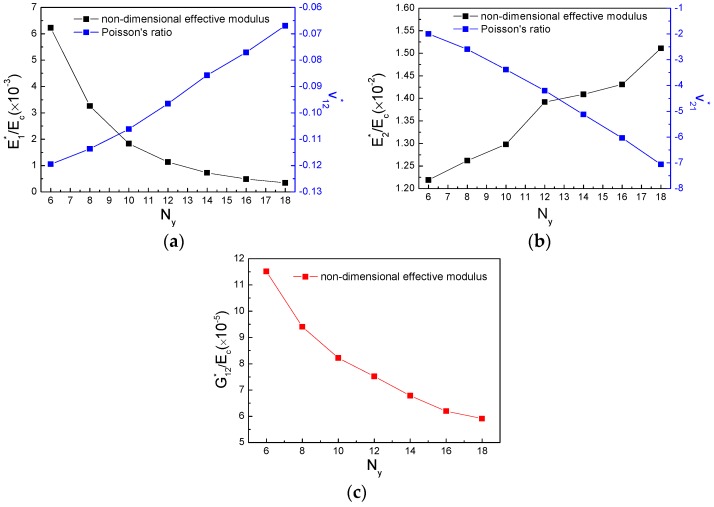
Variation of subordinate cell amount $N_{y}$ verse: (**a**) $E_{1}^{*}/E_{c}$ and Poisson’s ratio $\nu_{12}^{*}$, (**b**) $E_{2}^{*}/E_{c}$ and Poisson’s ratio $\nu_{21}^{*}$, (**c**) $G_{12}^{*}/E_{c}$.

The amount of star subordinate cells along x and y directions are defined and presented in Figure 8, where $N_{x}$ is the number of half star subordinate cell along x direction and $N_{y}$ is the number of entire star subordinate cell along y direction. The effects of subordinate cell amount on effective mechanical behavior were then studied. It may be clearly observed from Figure 9a that a growing amount of $N_{x}$ increases $E_{1}^{*}/E_{c}$ and $\nu_{12}^{*}$, when $N_{x}\geq 7$, $\nu_{12}^{*}$ turns positive. However, increasing $N_{x}$ makes both $E_{2}^{*}/E_{c}$ and $G_{12}^{*}/E_{c}$ decline and remains $\nu_{21}^{*}$ to be constant, presented in Figure 9b,c, respectively. Figure 10 shows how the effective properties of the new metamaterials vary with subordinate cell amount along y direction. Non-dimensional effective modulus $E_{1}^{*}/E_{c}$ and $G_{12}^{*}/E_{c}$ decrease with the increasing number of $N_{y}$, Poisson’s ratio $\nu_{12}^{*}$ increases and stays auxetic, whereas, $E_{2}^{*}/E_{c}$ and $\nu_{21}^{*}$ exhibit the opposite variations under an increasing number of $N_{y}$. Consequently, it is found that a small amount of $N_{x}$ and appropriate number of $N_{y}$ can satisfy the new hierarchical metamaterial with wholly auxetic behavior and strong stiffness in-plane simultaneously.

### 3.3. Relative Density Study

**Figure 11:**
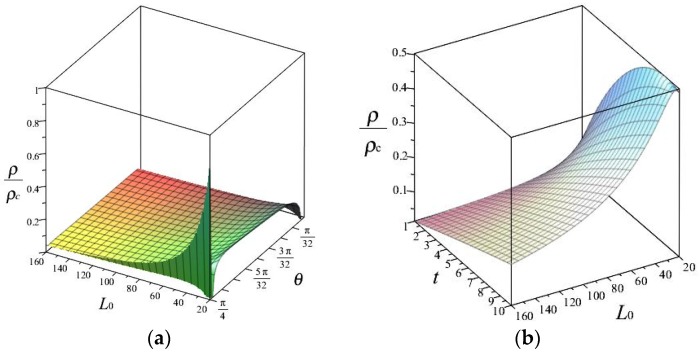

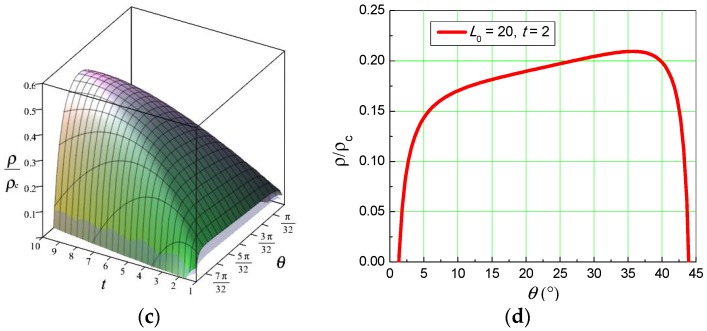
Geometric parameters effects on relative density of the hierarchical RUC: (**a**) t=2, (**b**) $\theta =30^{\circ }$, (**c**) $L_{0}=20$, (**d**) $L_{0}=20$ and t=2.

The formula of the relative density for one representative unit cell is given by Equation (2); the variation of relative density $\rho /\rho_{c}$ with parameters $L_{0}$,θ and t were obtained and are shown in Figure 11a–c. For Figure 11d, parameter θ was valid in a range of $2.5^{\circ }-42.5^{\circ }$, corresponding $\rho /\rho_{c}$ exhibits a non-monotonic going up and down variation. Therefore, the values of θ in the range of $35^{\circ }$ to $40^{\circ }$ made a different influence on the results of relative density as well as previous effective mechanical properties shown in Figure 7.

**Figure 12:**
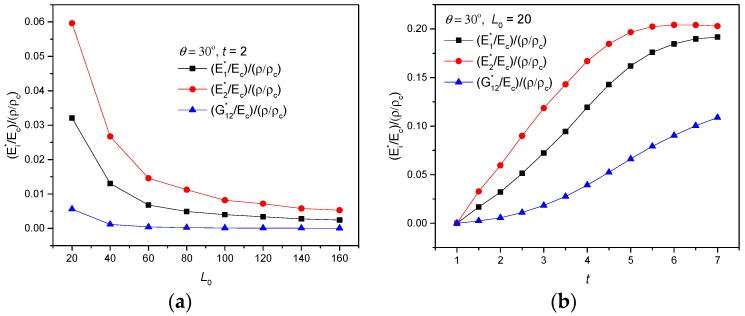

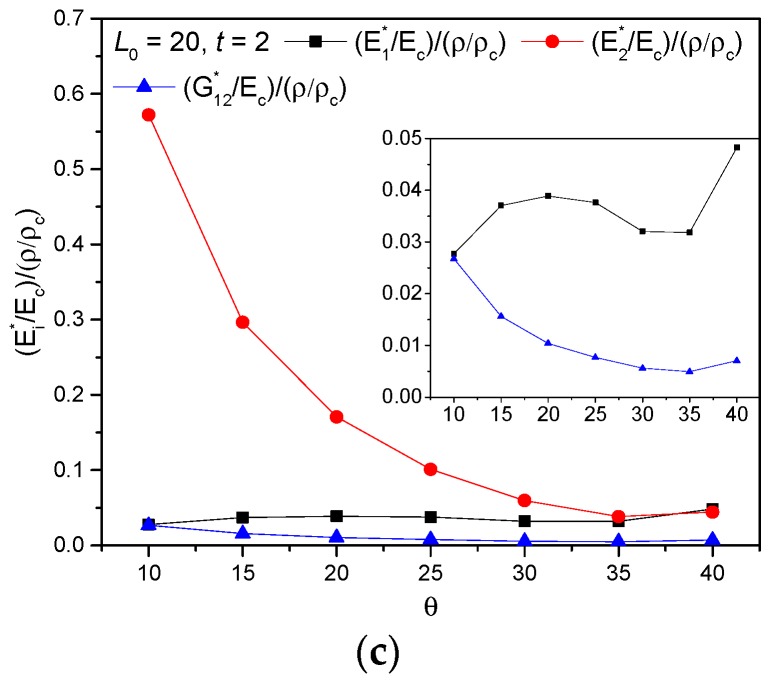
Variation of specific stiffness $(E_{i}^{*}/E_{c})/\left(\rho /\rho_{c}\right)$ with parameters: (**a**) $L_{0}$, (**b**) t, (**c**) θ.

**Figure 13:**
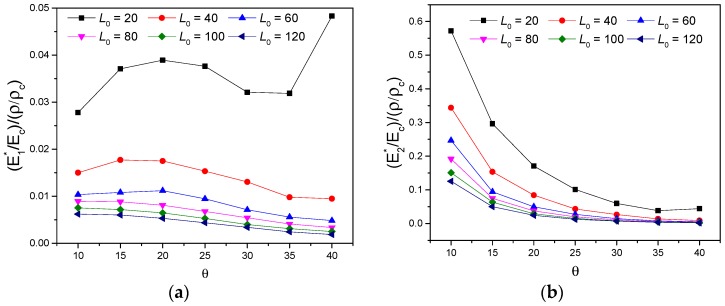

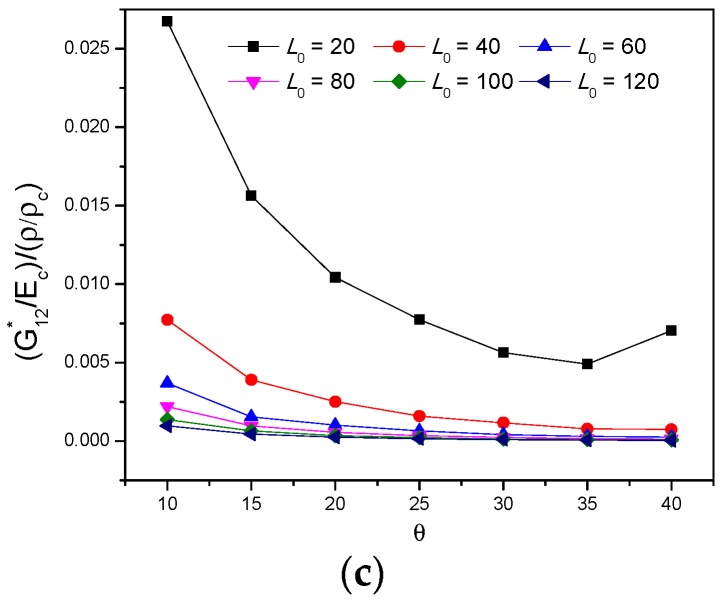
Variations of specific stiffness $(E_{i}^{*}/E_{c})/\left(\rho /\rho_{c}\right)$ verse parameters $L_{0}$ and θ, when t=2: (**a**) $(E_{1}^{*}/E_{c})/\left(\rho /\rho_{c}\right)$, (**b**) $(E_{2}^{*}/E_{c})/\left(\rho /\rho_{c}\right)$, (**c**) $(G_{12}^{*}/E_{c})/\left(\rho /\rho_{c}\right)$.

The relationship between effective mechanical properties and relative density $\rho /\rho_{c}$ simulated by FEM finite element method were investigated as following. Variations of specific stiffness $(E_{i}^{*}/E_{c})/\left(\rho /\rho_{c}\right)$ with different parameters are represented in Figure 12 and Figure 13, respectively. In Figure 12a, specific stiffness all decline with increasing $L_{0}$, extremum values of $(E_{2}^{*}/E_{c})/\left(\rho /\rho_{c}\right)=0.05963$ and $(G_{12}^{*}/E_{c})/\left(\rho /\rho_{c}\right)=0.00563$ were achieved with $L_{0}=20$, $\theta {=30}^{\circ }$ and t=2. Similarly, for the increase of cell thickness t, specific stiffnesses all exhibit a growing variation with different gradient, seen in Figure 12b. The impact of parameter θ on effective mechanical behavior of the proposed new metamaterial were investigated and are represented in Figure 12c. For the increase of θ, it may be observed that $(E_{2}^{*}/E_{c})/\left(\rho /\rho_{c}\right)$ and $(G_{12}^{*}/E_{c})/\left(\rho /\rho_{c}\right)$ have a resembling variation of first decline and then ascent, while $(E_{1}^{*}/E_{c})/\left(\rho /\rho_{c}\right)$ exhibit an up-down-up variation. In the range of $\theta {=35}^{\circ }$ to $\theta {=40}^{\circ }$, specific stiffnesses $(E_{i}^{*}/E_{c})/\left(\rho /\rho_{c}\right)$ all increase monotonically.

Figure 13 presents the variation of in-plane specific stiffness with the cell angle θ for various parameter $L_{0}$, while t=2. The specific stiffness along 1-direction exhibits three different variational trends with a rising θ for different $L_{0}$, respectively, shown in Figure 13a: (1) for $L_{0}=20$, $(E_{1}^{*}/E_{c})/\left(\rho /\rho_{c}\right)$ varies as an up-down-up curve and reaches the maximum value of 0.04829 with $\theta =40^{\circ }$, which is 25 times greater than the one for $L_{0}=120$; (2) for $L_{0}=40,60$, $(E_{1}^{*}/E_{c})/\left(\rho /\rho_{c}\right)$ presents a first increasing and then decreasing variation; (3) for $L_{0}\geq 80$, $(E_{1}^{*}/E_{c})/\left(\rho /\rho_{c}\right)$ decreases monotonically. From Figure 13b,c, it can be observed that $(E_{2}^{*}/E_{c})/\left(\rho /\rho_{c}\right)$ and $(G_{12}^{*}/E_{c})/\left(\rho /\rho_{c}\right)$ decrease clearly and then ascend with the variation of cell angle θ from $35^{\circ }$ to $40^{\circ }$ when $L_{0}=20$, a comparison of the specific stiffness in this range shows that $(E_{2}^{*}/E_{c})/\left(\rho /\rho_{c}\right)$ varies slightly from 0.03817 to 0.04422, however, $(G_{12}^{*}/E_{c})/\left(\rho /\rho_{c}\right)$ increases significantly from 0.0049 to 0.00704. When $L_{0}\geq 40$, $(E_{2}^{*}/E_{c})/\left(\rho /\rho_{c}\right)$ and $(G_{12}^{*}/E_{c})/\left(\rho /\rho_{c}\right)$ both monotonically decline with increasing θ and $L_{0}$. Figure 13c illustrates that an increase of more than 143% of the specific shear stiffness show up when the parameter $L_{0}$ varies from 20 to 120 with $\theta =40^{\circ }$, which makes varying $L_{0}$ also a good design method for $(G_{12}^{*}/E_{c})/\left(\rho /\rho_{c}\right)$. Therefore, it can be determined that all the high specific stiffness in plane can be achieved simultaneously by choosing proper parameter θ and $L_{0}$.

**Figure 14:**
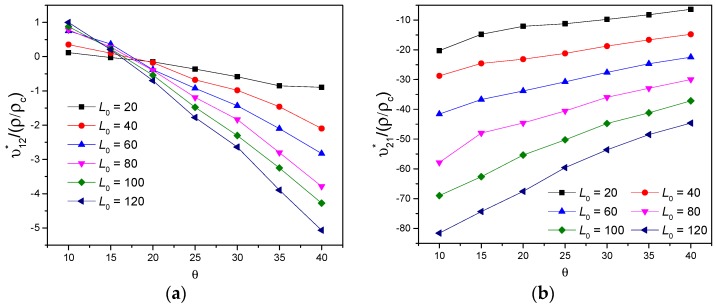
Variations of the ratio between effective Poisson’s ratio and relative density verse parameters $L_{0}$ and θ, when t=2: (**a**) $\upsilon_{12}^{*}/\left(\rho /\rho_{c}\right)$, (**b**) $\upsilon_{21}^{*}/\left(\rho /\rho_{c}\right)$.

Finally, the ratio between effective Poisson’s ratio and relative density verse parameters $L_{0}$ and θ were investigated. In Figure 14a, it is seen that $\upsilon_{12}^{*}/\left(\rho /\rho_{c}\right)$ performs from positive to remarkable auxetic behavior and it declines significantly with both increasing $L_{0}$ and θ, the maximal descending slope is achieved with $L_{0}=120$. Comparing with Figure 13a, the optimum values of both high specific stiffness and large auxetic deformation in 1-direction can be selected widely for the special curve shape of $(E_{1}^{*}/E_{c})/\left(\rho /\rho_{c}\right)$ with $L_{0}=20$. Figure 14b reveals that the auxetic $\upsilon_{21}^{*}/\left(\rho /\rho_{c}\right)$ increases with greater θ and decreases with increasing $L_{0}$, oppositely. Contrast to Figure 13b, the optimal values of both high specific stiffness and large auxetic deformation in 2-direction is acquired for $\theta =10^{\circ }$ and $L_{0}=20$, where $(E_{2}^{*}/E_{c})/\left(\rho /\rho_{c}\right)=0.57228$ and $\upsilon_{21}^{*}=-3.45649$.

## 4. Conclusions

A novel hierarchical metamaterial with tailorable mechanical properties was proposed using re-entrant planar lattice structure with star-shaped subordinate cell. The effective non-dimensional moduli and Poisson’s ratio in plane were simulated by FE homogenization firstly, then the influences of the geometric parameters on mechanical behavior and relative density were studied in detail. It was found that the new hierarchical metamaterial can obtain large variations and control of the design of the in-plane mechanics through the variations of parameters for both the re-entrant RUC and star subordinate cell. Comparing with conventional re-entrant honeycomb, the novel star-re-entrant metamaterial has enhanced mechanical properties of specific stiffness and auxeticity accounting for its hierarchical porosity as well as multilevel tunable parameters. In addition, the new auxetic metamaterial is more convenient fabricated by 3D printing technique as less stress concertation occurs in the connecting tips of star subordinate cell when compared with other zero Poisson’s ratio star cellular structure. However, the inclined angle of parent re-entrant RUC is restricted to 45° due to the symmetric simplification of star subordinate cell; as a result, in-plane effective mechanical behavior of the new hierarchical metamaterial can be limited by lacking another internal inclined angle of sub cell. In general, optimum results such as small values of parameters $L_{0}$, t and θ for sub cell as well as small amount of $N_{x}$ and moderate number of $N_{y}$ for parent RUC can provide the new hierarchical metamaterial with whole auxetic behavior and strong specific stiffness in-plane simultaneously. It is believed that the innovative hierarchical metamaterials will greatly expand the potential applications in the construction, manufacturing and transportation industries due to the inherent low-weight associated with hierarchical systems, like doubly curved panels in aerospace or marine structures. It can also be used in conformable and stretchable electronics, biomedical devices such as porous smart bandage releasing different classes of medications to different extents, as well as the design of smart auxetic stents, etc.

## References

[1] Gibson L.J., Ashby M.F. (1997). Cellular Solids: Structure and Properties.

[2] Ashby M.F. (2006). The properties of foams and lattices. Philos. Trans. R. Soc. Lond. Ser. A Math. Phys. Eng. Sci..

[3] Harkati E., Daoudi N., Bezazi A., Haddad F. (2017). Scarpa. In-plane elasticity of a multi re-entrant auxetic honeycomb. Compos. Struct..

[4] Lira C., Innocenti P., Scarpa F. (2009). Transverse elastic shear of auxetic multi re-entrant honeycombs. Compos. Struct..

[5] Scarpa F., Tomlinson G. (2000). Theoretical characteristics of the vibration of sandwich plates with in-plane negative Poisson’s ratio values. J. Sound Vib..

[6] Liu W., Wang N., Luo T., Lin Z. (2016). In-plane dynamic crushing of re-entrant auxetic cellular structure. Mater. Des..

[7] Innocenti P., Scarpa F. (2009). Thermal conductivity properties and heat transfer analysis of multi-re-entrant auxetic honeycomb structures. J. Compos. Mater..

[8] Lakes R. (1983). Materials with structural hierarchy. Nature.

[9] Oftadeh R., Haghpanah B., Papadopoulos J., Hamouda A.M.S., Nayeb-Hashemi H., Vaziri A. (2014). Mechanics of anisotropic hierarchical honeycombs. Int. J. Mech. Sci..

[10] Mousanezhad D., Ebrahimi H., Haghpanah B., Ghosh R., Ajdari A., Hamouda A.M.S., Vaziri A. (2015). Spiderweb honeycombs. Int. J. Solids Struct..

[11] D’Alessandro L., Zega V., Ardito R., Corigliano A. (2018). 3D auxetic single material periodic structure with ultra-wide tunable bandgap. Sci. Rep..

[12] Bruggi M., Corigliano A. (2019). Optimal 2D auxetic micro-structures with band gap. Meccanica.

[13] Wang Q.M., Jackson J.A., Ge Q., Hopkins J.B., Spadaccini C.M., Fang N.X. (2016). Lightweight mechanical metamaterials with tunable negative thermal expansion. Phys. Rev. Lett..

[14] Billon K., Zampetakis I., Scarpa F., Ouisse M., Hetherington A. (2017). Mechanics and band gaps in hierarchical auxetic rectangular perforated composite metamaterials. Compos. Struct..

[15] Qiao J., Chen C. (2016). In-plane crushing of a hierarchical honeycomb. Int. J. Solids Struct..

[16] Sun Y., Chen Q., Pugno N. (2014). Elastic and transport properties of the tailorable multifunctional hierarchical honeycombs. Compos. Struct..

[17] Sun Y., Wang B., Pugno N., Wang B., Ding Q. (2015). In-plane stiffness of the anisotropic multifunctional hierarchical honeycombs. Compos. Struct..

[18] Taylor C.M., Smith C.W., Miller W., Evans K.E. (2011). The effects of hierarchy on the in-plane elastic properties of honeycombs. Int. J. Solids Struct..

[19] Mousanezhad D., Haghpanah B., Ghosh R., Hamouda A.M., Nayeb-Hashemi H., Vaziri A. (2016). Elastic properties of chiral, anti-chiral, and hierarchical honeycombs: A simple energy-based approach. Theor. Appl. Mech. Lett..

[20] Gatt R., Mizzi L., Azzopardi J.I., Azzopardi K.M., Attard D., Casha A., Briffa J., Grima J.N. (2015). Hierarchical Auxetic Mechanical Metamaterials. Sci. Rep..

[21] Chen Y., Jia Z., Wang L. (2016). Hierarchical honeycomb lattice metamaterials with improved thermal resistance and mechanical properties. Compos. Struct..

[22] Yin H., Huang X., Scarpa F., Wen G., Chen Y., Zhang C. (2018). In-plane crashworthiness of bio-inspired hierarchical honeycombs. Compos. Struct..

[23] Wu W., Tao Y., Xia Y., Chen J., Lei H., Sun L., Fang D. (2017). Mechanical properties of hierarchical anti-tetrachiral metamaterials. Extre. Mech. Lett..

[24] Wu W., Song X., Liang J., Xia R., Qian G., Fang D. (2018). Mechanical properties of anti-tetrachiral auxetic stents. Compos. Struct..

[25] Gong X., Huang J., Scarpa F., Liu Y., Leng J. (2015). Zero Poisson’s ratio cellular structure for two-dimensional morphing applications. Compos. Struct..

[26] Lira C., Scarpa F., Olszewska M., Celuch M. (2009). The SILICOMB cellular structure: Mechanical and dielectric properties. Phys. Status Solidi.

[27] Salit V., Weller T. (2009). On the feasibility of introducing auxetic behavior into thin-walled structures. Acta Mater..

[28] Zhang W., Neville R., Zhang D., Scarpa F., Wang L., Lakes R. (2018). The two-dimensional elasticity of a chiral hinge lattice metamaterial. Int. J. Solids Struct..

